# Should Adherence to Medical Recommendations Be a Requirement for Kidney Transplant Candidacy?

**DOI:** 10.1080/15265161.2026.2691943

**Published:** 2026-06-29

**Authors:** Catherine R. Butler, Aaron G. Wightman

**Affiliations:** a University of Washington; b Veterans Affairs Puget Sound Health Care System; c Seattle Children’s Research Institute

**Keywords:** Organ transplantation, rationing/resource allocation, health policy

## Abstract

To be considered a candidate for transplant, patients with advanced kidney disease must demonstrate their ability to adhere to medical recommendations. an adherence criterion is intended to ensure effective use of deceased donor kidneys by selecting candidates who are able to engage in complicated post-transplant clinical care. However, evidence that pre-transplant assessment of adherence predicts meaningful post-transplant outcomes is equivocal. Further, this criterion risks exacerbating existing inequities in access to transplant and rigid definitions of what constitutes adherent behavior may impede opportunities to tailor treatment plans to individual preferences and circumstances. It is also unclear that an adherence criterion supports ethical standards for a fair process of resource allocation including transparency, relevance to affected groups, regulation, and opportunity for revision. We offer several strategies to revise the current approach to evaluating transplant candidates for adherence to better align with ethical principles guiding allocation of a scarce societal resource.

## INTRODUCTION

For many people with kidney failure, kidney transplant offers an opportunity for greater longevity and quality of life compared with dialysis treatment (Rao et al. 2007; Purnell et al. 2013; Harhay et al. 2020). However, 136,000 people develop kidney failure in the US each year and only about 26,000 receive a transplant (United States Renal Data System 2023). In order to receive a kidney, patients with advanced kidney disease or kidney failure must be referred to a transplant center, complete a physical and psychosocial evaluation, and be selected as a candidate by a transplant center team. A patient can then be listed on the national deceased donor waitlist and/or work to identify a living donor.

Patients who are not able to demonstrate sufficiently adherent behavior during the kidney transplant evaluation process may be deferred or declined as transplant candidates. Indeed, by one estimate, nonadherent behavior is cited as a reason for declined candidacy among 28% of patients evaluated at a transplant center (Holley et al. 1998) (though practice patterns likely vary over time and between centers (Morenz et al. 2026)). In a survey study of transplant centers, over half ranked non-adherence as one of the top barriers to completing the evaluation process (Buford et al. 2026). Post-transplant care involves a complex regimen of medications and regular clinical follow-up throughout a transplant recipient’s life. For this reason, a patient’s ability to adhere to a treatment plan is important for both their own health and safety as well as effective use of scarce donor organs. Non-adherence to immunosuppressant medications can lead to kidney loss, need for a second kidney (further taxing a scarce resource) and/or immunological sensitization that prohibits a person from receiving a second transplant. However, in all but severe cases of inability to engage in medical care, patients with imperfect adherence may still stand to personally benefit from transplant compared with alternative treatment options. Estimated risks of transplant kidney loss, even for those with imperfect adherence to medications, still compare favorably with the approximately 18% annual risk of death for patients remaining on diaysis (United States Renal Data System 2025). It is common for medical treatment options to entail risks but also potential benefits to a patient and a decision is typically approached through shared decision-making. However, kidney transplant candidacy is frequently unilaterally declined or deferred because of concerns about adherence despite transplant being a desired and potentially beneficial option for an individual patient. In these cases, an adherence criterion is better understood as a strategy for resource allocation rather than a way of supporting an individual patient’s best interests.

The Organ Procurement and Transplantation Network (OPTN), a public-private partnership that oversees transplant activities across the US, affirms three ethical principles that guide appropriate transplant allocation practices: utility, equity (also referred to as ‘justice’), and respect for patient autonomy (HRSA 2018). The ethical foundations of any resource allocation process can also be assessed in reference to features of a “fair process”, including transparency of the process, relevance of selection criteria to affected persons or communities, and opportunity to appeal, revise, and regulate the process (Daniels and Sabin 2008). To align with these principles, the current system of allocation of deceased donor kidneys among patients on the national waitlist is governed by a set of standardized and published rules (Stegall et al. 2017). These rules have undergone rigorous ethical analysis, community deliberation, and revision over decades of refinement. Far less attention has been paid to upstream processes of candidate evaluation and selection—including assessment of patient adherence—but recognition of inefficiencies and disparities in access to the kidney transplant waitlist increasingly call attention to these upstream processes (Berry et al. 2019). We herein analyze the ethical underpinnings of an adherence criterion for kidney transplant candidacy.

### CURRENT PRACTICES AROUND ADHERENCE AS A CRITERION FOR KIDNEY TRANSPLANT CANDIDACY

Assessment of a patient’s ability to adhere to clinical recommendations is a standard part of the kidney transplant psychosocial evaluation, required by the Centers for Medicare and Medicaid Services (Holley et al. 1998; Tong et al. 2011; Code of Federal Regulations 2019). Clinical practice guidelines from professional societies further endorse the need to evaluate adherence behaviors among transplant candidates primarily as an opportunity to support individual patients by identifying and addressing psychosocial barriers to engagement in post-transplant care (OPTN Ethics Committee 2023). Several prominent guideline statements suggest that only in extreme circumstances of “life-threatening” or “health-compromising” nonadherent behavior should nonadherence justify excluding transplant candidacy (Kasiske et al. 2001; Chadban et al. 2020). Other practice guidelines do include broader statements that “nonadherence to therapy is a contraindication to kidney transplant” (Knoll et al. 2005).

While many kidney transplant centers do not publish specific candidacy criteria, surveys and qualitative work suggest that in practice, concerns around a range of degrees and types of nonadherence are common reasons for deferring and/or declining patients as transplant candidates (Holley et al. 1998; Butler et al. 2021). Several survey measures have been designed to represent psychosocial risk factors, including adherence behaviors, but use of these standardized tools is inconsistent across clinicians and transplant centers (Puttarajappa et al. 2021; OPTN Ethics Committee 2023; Obayemi et al. 2025). Assessments of adherence to recommended health behaviors and prescribed treatments before transplant, such as weight loss, exercise, diet, medication management, and dialysis sessions, are often used as surrogate markers of how well a patient is likely to adhere to treatment recommendations after transplant (Holley et al. 1998). For example, blood phosphorus level may be used as an indicator of adherence to medications (i.e., phosphate binders) and dietary recommendations. Regular attendance, cooperative behavior, and completion of dialysis sessions is also commonly reviewed by transplant center teams. Qualitative work suggests that assessment of adherence can sometimes expand beyond these aspects of kidney care to include judgments about a range of other health and social behaviors (Butler et al. 2021). A patient’s ability to navigate the transplant evaluation process itself—which can be complex, opaque, and prolonged—may be considered a litmus test of their ability to engage in clinical care after transplant (Butler et al. 2020, 2025).

### UTILITY OF KIDNEY TRANSPLANT CANDIDATE SELECTION BASED ON ADHERENCE

A central goal of transplant candidate selection is to maximize aggregate utility, or benefit, of a limited supply of deceased donor kidneys. Transplant centers select recipients who are sufficiently likely to benefit in terms of their own longevity and/or the survival of a donor kidney. Early loss of a kidney transplant benefits neither the recipient, the transplant center, nor the organ allocation system as a whole and nonadherence to immunosuppression medications is recognized to be a leading cause of kidney transplant failure (De Geest et al. 1995; Hilbrands et al. 1995). For these reasons, there is a strong intuitive argument for ensuring that transplant recipients will be able to adhere to clinical recommendations and maintain close clinical follow-up throughout their lifetimes. However, the value of an adherence criterion in maximizing utility in kidney transplant allocation is predicated on 2 assumptions: (1) there is a sufficiently strong and consistent link between pre-transplant adherence and post-transplant behaviors and (2) post-transplant adherence is sufficiently predictive of meaningful patient outcomes and graft survival.

Pre-transplant characteristics and behaviors are often used as surrogate markers for expected post-transplant adherence. While the assumption that past behavior predicts future behavior is intuitively appealing, there are many reasons that patterns of adherence may change after a kidney transplant. Life on dialysis is categorically different than life with a kidney transplant and reasons for skipped or shortened dialysis sessions—such as caregiving responsibilities, pain, and fatigue—may not constitute barriers to consistent medication adherence after transplant. The substantial shift in life experience inherent to receiving a transplant, including its impact on quality of life, burden of treatment requirements, and social and psychological wellbeing, might reasonably be expected to transform health behaviors (Purnell et al. 2013). Evidence to support the reliability and strength of the link between past behaviors and post-transplant adherence is mixed. For example, there is conflicting evidence from retrospective studies about whether and how younger age, substance use, shortened dialysis sessions, and intradialytic weight gain are associated with post-transplant nonadherence (Hilbrands et al. 1995; Bosma et al. 2011; Sandhu et al. 2011; Hartono et al. 2017; Paterson et al. 2018). Medication adherence behaviors can be difficult to capture (Foster et al. 2018), but survey-based studies suggest that self-reported medication adherence is associated with episodes of transplant rejection (Dobbels et al. 2009). Pre-transplant high phosphate level (an indirect measure of nonadherence to phosphate binder medications and dietary recommendations) has also been linked to post-transplant nonadherence in several studies (Hucker et al. 2019; Kitamura et al. 2019; Sawinski et al. 2022).

The value of selecting transplant candidates who will be adherent to treatment recommendations also requires that post-transplant adherence behaviors are sufficiently predictive of meaningful patient outcomes. Existing studies suggest that imperfect adherence for even 5% of a transplant recipient’s immunosuppressant dosing schedule is associated with increased risk of donor kidney loss (Nevins and Thomas 2009; Nevins et al. 2017). However, despite how common imperfect adherence to medications can be among transplant recipients—by one estimate, 50% of transplant recipients had at least one concern about adherence to immunosuppressive medications in a 3-month period (Yoon et al. 2022)—overall graft survival rates remain excellent. Sawinski and colleagues found that pre-transplant hyperphosphatemia was associated with a 27% increased risk of kidney failure in the year after transplant (Sawinski et al. 2022). However, these relative risks should be interpreted in the context of what are increasingly positive outcomes for kidney transplant recipients. In 2020, 1-year graft survival was approximately 92% for deceased donor kidney transplants (United States Renal Data System 2023). A 27% higher relative risk of transplant failure would then confer an absolute risk difference of roughly 2% 1-year transplant loss (that is, 10% 1-year graft loss for those with pre-transplant hyperphosphatemia vs 8% for those without). It is possible that the effects of a moderate degree of medication nonadherence become more apparent in shaping longer-term outcomes, but there are few long-term studies available to inform this hypothesis. In sum, while adherence to post-transplant medications is to be encouraged, the great majority of transplant recipients with imperfect adherence will not experience graft failure and adherence behaviors may not be sufficiently predictive of this outcome to justify excluding patients from a highly beneficial treatment.

### EQUITY IN KIDNEY TRANSPLANT CANDIDATE SELECTION BASED ON ADHERENCE

The principle of equity requires that societal benefits and burdens be evenly distributed across a population. Similar respect and consideration should be extended to all people in need of a scarce societal resource, such as deceased donor kidneys, and access should not be shaped by factors such as race, ethnicity, socioeconomic status, or gender (Gutmann 1983). However, there are deep and persistent disparities in access to kidney transplant for minoritized racial groups and other socially disadvantaged populations (Epstein et al. 2000; Patzer et al. 2015; Wesselman et al. 2021). Optimally, criteria for transplant candidate selection would reduce these disparities, and at minimum, these criteria should avoid directly or indirectly exacerbating disparities (Obayemi et al. 2025).

Adherence behaviors are closely tied to multiple domains of social determinants of health (Sharp 1999; Sabate 2003; OPTN Ethics Committee 2023). For example, patients’ ability and willingness to take recommended medications may be shaped by a wide range of factors including pill burden and side effects, cost and barriers to acquiring prescriptions, and trust in providers (Sharp 1999). Nonadherence to complex dialysis regimens may reflect even more diverse personal and social factors including complexity of treatment regimens, inadequate education, and burden of illness. Through these pathways, an adherence criterion for transplant candidacy risks indirectly exacerbating existing health disparities (Kilner 1990). Indeed, psychosocial assessments for transplant candidacy have been shown to disproportionately exclude minoritized populations (Deutsch-Link et al. 2023).

Because many guidelines around assessment of adherence are vague or nonspecific, there is substantial heterogeneity in approach to this assessment between transplant centers and across clinicians (Akolekar et al. 2008; Mandelbrot et al. 2017; Shpigel et al. 2019). This means that similar patients may be treated differently for arbitrary reasons such as where they live or which clinician they happen to encounter (Kilner 1988; Levenson and Olbrisch 1993). Further, socioeconomically disadvantaged groups tend to experience negative cultural stereotypes and may be disproportionately impacted by clinicians’ implicit biases and resulting judgments of what constitutes adherence (Chapman et al. 2013; National Academies of Sciences, Engineering, and Medicine 2022). In other healthcare contexts, minoritized patients have described feeling forced to adjust behaviors and present as compliant in order to receive necessary care (Mattingly 2018). The overt nature of adherence assessments and the highly consequential implications of transplant evaluation might be expected to magnify this power differential.

Behavioral patterns, such as nonadherence, are commonly considered to be under the control of a patient and therefore their personal responsibility. Participants in a public survey selected against a hypothetical transplant candidate with nonadherent behavior in favor of someone with a more physiological risk factor that the patient could not control, such as a type of kidney disease that is likely to recur after transplant (Drezga-Kleiminger et al. 2025). Adolescent populations of kidney transplant recipients demonstrate higher rates of nonadherence, but there is significant hesitation to exclude these patients from transplant, perhaps in part, because we attribute less personal responsibility to pediatric patients. However, better appreciation of the complex ways in which social determinants of health may shape a patient’s behaviors and limit options makes it challenging to directly attribute responsibility for these behaviors. Further, personal responsibility, or blame, for a health condition is not typically considered to be an acceptable reason for denying beneficial care. Rather, a principle of equity is supported by an assumption that all patients are similarly deserving of a scarce healthcare resource.

### RESPECT FOR PATIENT AUTONOMY IN SELECTION BASED ON ADHERENCE

The scarcity of deceased donor kidneys means that not all patients who wish for and would benefit from a kidney transplant can receive one. That is, a system of resource allocation may necessarily impinge upon individual autonomy in order to prioritize effective and equitable allocation of donor kidneys across the population. However, even within these constraints on patient choice, there are opportunities to support the autonomy and dignity of individual patients engaged in the evaluation process (Gordon et al. 2013; Rosas and Reid 2025). The OPTN principles for organ allocation specify that respect for individual autonomy requires “transparency of processes and allocation rules to enable stakeholders to make informed decisions.” The transplant evaluation process can have far reaching implications for decisions related to kidney failure treatments, other health conditions, and life activities more broadly (Butler et al. 2025). A requirement that patients demonstrate strict adherence to standard treatment approaches may limit the opportunity of patients and their local clinicians to tailor and individualize care—both within and outside the transplant process—to align with patient priorities, values, and individual context.

The term “adherence” is intended to describe health behaviors that align with shared treatment decisions made by a patient and their clinician (Holm 1993; Sabate 2003). This definition contrasts with the more paternalistic concept of “compliance,” which connotates a passive following of treatment recommendations. However, it is not clear that a person-centered definition of adherence is consistently operationalized in practice (Allen et al. 2011). An imperative to demonstrate adherence in the kidney transplant evaluation process may encourage rigid application of check-list or standardized approaches to treatment that can conflict with principles of person-centered care (Butler et al. 2021). Further, the desire to receive a kidney can serve as a strong incentive to comply with any and all requirements for transplant candidacy (Silva et al. 2014), even if these are not what a patient would choose in the course of usual care. Assessment of adherence and surveillance over health behaviors can have far-reaching implications for care decisions on a range of other health conditions (Holley et al. 1998).

Americans with kidney failure are increasingly older and have a high burden of comorbid health conditions (Tonelli et al. 2018; United States Renal Data System 2023). An algorithmic application of tests and treatments based on clinical practice guidelines may be especially burdensome for this population (Boyd et al. 2005). For example, an aggressive approach to screening for asymptomatic conditions such as prostate or breast cancer may not improve outcomes for older adults and can lead to false positive results, extra testing, and complications (Wilt et al. 2008; Walter and Schonberg 2014). For these reasons, shared decision-making is increasingly accepted as an approach to tailor care to individual values and clinical context (Walter and Covinsky 2001; Barry and Edgman-Levitan 2012). However, a patient pursuing kidney transplant may feel obligated to complete any and all recommended tests and treatments in order to be considered a candidate. Primary care and local kidney providers might be positioned to help patients interpret requirements of the transplant evaluation and determine what adherence means in a patients’ individual context. However, because practices and candidacy criteria vary between transplant teams and there is rarely explicit documentation of how adherence will be judged, local providers often have limited insight into the evaluation process (Butler et al. 2020).

### PROCEDURAL JUSTICE AROUND ADHERENCE AS A SELECTION CRITERION

Another approach to evaluating the ethical foundation of a process of scarce resource allocation is to consider whether the process upholds features of procedural justice. A turn to procedural justice is especially valuable when directives from other principles of selection (i.e., utility, equity, respect for patient autonomy) are indeterminant or conflicting. A “fair process” of selection is transparent, includes selection criteria that are agreed upon by those who are subject to the process, is regulated, and is responsive to appeal and revision (Daniels and Sabin 2008).

There is question as to whether the broader system of transplant candidate selection can meet the criteria for a fair process. Few transplant centers publish explicit lists of criteria for candidacy—or how and why these criteria were chosen—and transplant committee deliberation is typically conducted confidentially with limited opportunity for appeal (Husain et al. 2024; Pawar et al. 2025). However, even if this broader transplant selection process were structured to align with criteria for a fair process, there remain questions as to whether an adherence criterion could itself meet these standards.

#### Transparency

A transparent approach to selection of candidates to receive a scarce public resource supports consistent application of guiding principles across cases and can engender public trust. Adherence is listed as a criterion for selection in clinical practice guidelines and qualitative work suggests that patients and their family members also recognize that their adherence to clinical recommendations will be monitored and contribute to their transplant candidacy (Sharp 2006; Butler et al. 2025). However, it is not clear that the manner in which an adherence criterion is applied to the transplant candidate selection process is entirely transparent. There is no commonly accepted definition or measure for adequate adherence. Assessment of adherence and implications of this assessment for transplant candidacy vary widely between transplant centers. This lack of standardization limits public dissemination of information around how adherence is used in transplant candidate selection.

#### Relevance to Affected Persons and Communities

When the directives of ethical principles guiding a system of allocation conflict, those persons who are affected by this system are best positioned to judge which goals should take priority. Kidney transplant allocation most directly affects patients and their family members. However, as currently configured, transplant center clinicians largely govern the approach to transplant candidate selection, including considerations around adherence criteria. Existing work suggests that clinicians tend to prioritize selection practices that maximize aggregate utility of a limited supply of donor organs (Sypek et al. 2022), though many do feel conflicted about the potential for inequity resulting from this approach (Tong et al. 2011). Transplant center quality metrics such as 1- and 3-year kidney graft survival further incentivize programs to be more conservative about selecting transplant candidates and more rigid in use of selection criteria, such as adherence.

While lay community values around organ allocation are complex and diverse (Tong et al. 2010; Gibbons et al. 2017), existing evidence from surveys, community engagement studies, and qualitative work suggest that the public may prioritize equity over utility in allocating a scarce healthcare resource (Nord et al. 1995; Ubel and Loewenstein 1996), including kidney transplants (Tong et al. 2010; Sypek et al. 2022). In qualitative work, people with kidney failure draw on their own difficult experiences of illness and emphasize the importance of giving everyone in need a similar chance to receive a kidney (Geddes et al. 2005; Tong et al. 2012). A discrete choice experiment suggested that community members would allocate donor kidneys in a way that respects a common need for transplant among all patients with kidney failure rather than focusing on maximizing life expectancy in recipient selection (Howard et al. 2015). In contrast, a study gathering public sentiment about the use of artificial intelligence in selecting liver transplant recipients suggested that respondents were most consistent in prioritizing factors such as urgency of need and likelihood of survival, but a majority also felt that predicted medication adherence should be considered in candidate selection (Drezga-Kleiminger et al. 2025). Collectively, this existing evidence suggests that public sentiment around an adherence criterion is complex, but does indicate that the patient community may have concerns around approaches to selection that impinge on equity and respect for individual autonomy.

#### Revision and Regulation

When selection criteria are transparent and standardized, it is possible to monitor, deliberate over, and improve an approach to transplant candidate selection (Baker 1993; Chapman et al. 2013). For example, selection among patients on the national deceased donor waitlist is largely driven by a set of published rules. Identification of inequities and inefficiencies in selection among patients on the transplant waitlist has prompted multiple cycles of revision and improvement over the last decades (Wilk et al. 2017; Zhang et al. 2018). However, lack of transparent or standardized processes around assessing adherence as a criterion for transplant candidacy means that there is little opportunity to monitor and improve upon this component of the transplant selection process.

### OPERATIONAL CONSIDERATIONS

Considerations outside the ethical principles of resource allocation also influence transplant candidate selection practices. Transplant center staff capacity is often strained, and teams may be concerned that accepting candidates without optimal adherence behaviors may create a greater workload in caring for these patients after transplant and detract from the care of other patients. Transplant centers may also encounter financial pressures to select patients who are likely to require more straight-forward evaluation processes and experience smooth post-transplant courses. While these contextual factors are outside the scope of an ethical analysis of transplant selection criteria, a system of allocation is unlikely to be successful in practice if these types of operational barriers are not also considered and addressed (Butler et al. 2024).

### RECONCILIATION

In summary, there are strong ethical concerns raised by current practices around an adherence criterion for kidney transplant candidacy. Persistent gaps in empirical evidence are unlikely to be addressed in the near term, and there may yet be residual ethical uncertainty when principles and priorities conflict. In this context, we propose three routes for adapting current use of an adherence criterion to better align with existing evidence and ethical foundations.

#### Solution 1: Forgo Assessment of Adherence

There is limited evidence to support the utility of pre-transplant adherence in predicting post-transplant outcomes, and there are concerns that an adherence criterion may not support equitable allocation, respect for patient autonomy, or fair process. Given these concerns, the clinical community could decide to forgo an adherence criterion for kidney transplant candidacy altogether. A formal evaluation of adherence might still be valuable as part of the preparation for kidney transplant to identify opportunities for additional education or other resources to enhance a recipient’s ability to manage post-transplant care (National Academies of Sciences, Engineering, and Medicine 2022). However, under this approach, nonadherence would not be used as a criterion to exclude transplant candidacy.

Strict exclusion of an adherence criterion in the context of kidney transplant candidate selection does risk reducing the aggregate benefit of the supply of deceased donor kidneys. For patients with irresolvable psychiatric or behavioral barriers to adherence, complete inability to take post-transplant medications inevitably leads to donor kidney immunologic rejection and kidney failure. In these cases, kidney transplant may entail greater risk for an individual than benefit. Nonetheless, there is an ethical and empirical precedent for this type of strict approach. During the COVID-19 pandemic, concerns for impending scarcity of mechanical ventilators prompted extensive deliberation among community members, clinicians, and ethicists around appropriate allocation of scarce resources (Ne’eman et al. 2021). A common element of many triage models was the utilization of a limited and standardized set of data items designed to avoid incorporating implicit biases into triage decisions. For example, a Delphi study among members of the disaster response clinical community resulted in general agreement that factors such as gender, race, body mass index, and social context should not be incorporated into triage processes even if these variable were hypothesized to offer incremental benefit in assessing prognosis (Gray et al. 2022). Similarly, a system of kidney transplant candidate selection might be designed to prioritize fairness by entirely excluding an adherence criterion even at the risk of less than maximal population-level outcomes.

#### Solution 2: Implement a Minimalist Adherence Criterion

Another strategy to implement an adherence criterion that better aligns with ethical principles would be to restrict the use of this criterion to extreme cases of nonadherent behavior. This approach also aligns more closely with existing clinical practice guidelines for transplant candidate selection that recommend that nonadherence should be used to exclude transplant candidates only in rare situations in which nonadherent behavior is irresolvable and life threatening (Kasiske et al. 2001; Chadban et al. 2020). Under a minimalist approach, assessment of adherence would focus on identifying and excluding patients who do not stand to personally benefit from kidney transplant as a treatment option because of major non-modifiable barriers to adherence. Under this minimalist approach, nonadherence would only be invoked as a contraindication to transplant if profound inability to follow treatment plans would mean that transplant would not be beneficial—and may even be harmful—for an individual.

While such a minimalist approach to an adherence criterion would benefit from additional empirical evidence and established standards to put into practice (Sawinski and Foley 2020), the approach does benefit from being analogous to other types of pre-operative clinical decision-making. Indeed, the broader practice of medicine is directed at identifying those interventions that would or would not provide overall benefit for a patient based on their individual situation. Over the course of training and practice, clinicians accumulate experience in applying this standard even in situations of uncertainty or ambiguity. For example, mechanical cardiac valve replacement can be lifesaving but carries high risk of infection for people with substance use disorder who are likely to resume use of intravenous drugs. Only in extreme and rare circumstances would a clinical team determine that risk of nonadherence to a plan for post-operative abstinence from drugs outweighs the potential benefit of a life-saving treatment for an individual patient (Kirkpatrick and Smith 2018). A test of whether a minimalist adherence criterion is being used appropriately would be to ask, “Would the patient be a transplant candidate if donor kidneys were not scarce?” or “Is kidney transplant in the patient’s best interests?”

An operational application of a minimalist approach to an adherence criterion would require a transplant center to adopt a more standardized and structured process of evaluation, deliberation, selection, and reporting of candidacy decisions. Such workflows would be designed to explicitly separate processes intended to determine transplant candidacy from those intended to identify ways to support patients in improving post-transplant outcomes. Such an explicit separation in clinical processes with categorically different goals (i.e., resource allocation vs person-centered care) would be broadly applicable to improving transparency in the kidney transplant selection process as a whole. The assessment of whether nonadherent behaviors might truly preclude a patient from benefiting from transplant (or any other treatment) is likely prone to bias. Explicit guidelines about what types and degrees of nonadherent behavior should constitute a contraindication to transplant, bias training, and empirical evidence about associations between nonadherence and post-transplant outcomes would be valuable in supporting a minimalist approach to an adherence criterion. Transplant centers might also institute a prohibition on discussing concerns about non-adherence in candidate selection meetings if these concerns to not constitute an absolute contraindication to transplant candidacy.

#### Solution 3: Implement a Standardized Adherence Assessment and Criterion

A third approach to reinforcing the ethical foundation for an adherence criterion for transplant candidacy would be to develop and universally implement a standardized measure or evaluation procedure. If adherence is to be used as a criterion for selecting recipients of a scarce resource among those who could all benefit, this criterion should be implemented in a way that is transparent, standardized, and accountable to patients, families, and communities. This approach would align with efforts to standardize and develop evidence-based criteria for other transplant candidacy requirements (Sawinski and Foley 2020; Kao et al. 2022).

Several standardized measures of psychosocial status have demonstrated mixed results in predicting post-transplant outcomes (Obayemi et al. 2025). The Stanford Integrated Psychosocial Assessment for Transplantation (SIPAT) is a standardized psychometric instrument designed to predict post-transplant risk and includes a domain to characterize adherence. Evidence that this tool can predict post-transplant adherence or other outcomes is mixed (Maldonado et al. 2015; Chen et al. 2019; Perry et al. 2024) and the tool has not been reliably associated with post-transplant survival (Maldonado et al. 2015; Chen et al. 2019). Further, SIPAT psychosocial domains are highly correlated with social determinants of health including marital status, educational attainment, and race (Chen et al. 2019). The “TAKE-IT” clinical trial measured adherence with a 30-day period of self-administered placebo pills monitored by smart pillboxes, which might serve as a model for a more direct test of health behaviors relevant to post-transplant outcomes (Foster et al. 2018). However, a high rate of incomplete electronic pillbox data capture during this trial suggests some operational challenges to this kind of approach.

Broad implementation of an evidence-based measure of adherence would require not only additional empirical confirmation of validity, but also deliberation and agreement among community members, clinicians, and ethicists about how such a tool would be applied in practice. A consensus around how to apply a standardized measure of adherence would require balancing values to support most efficient use of scarce donor organs, equity in access to treatment, and respect for patient autonomy. An intentional process of community deliberation could help to identify an acceptable balance between these potentially conflicting values (Blacksher et al. 2012; Goold et al. 2012). The real-world effect of a standardize adherence criterion could then be monitored, evaluated, and revised over time to ensure that the approach supports intended community values and priorities.

Historically, efforts to explicitly ration life-saving medical treatments, especially when these seem to impinge on equity, have resulted in deep disagreements within and between public and clinical communities. For example, attempts to develop a system of explicit allocation of life-saving resources during the COVID-19 pandemic were intended to make best use of scarce ventilator machines in a tragic situation (Sprung et al. 2020). However, the fairness of these rationing protocols was heavily criticized and they were rarely put into practice despite extreme resource scarcity (Ne’eman et al. 2021). Well-intentioned attempts to explicitly ration Oregon Medicaid services were ultimately abandoned because no solution was without significant drawbacks (Dixon and Welch 1991). These experiences prompt some to question whether standardized and transparent systems of rationing may ever be publicly palatable (Dixon and Welch 1991; Ubel 2007). Withholding a life-saving resource is inevitably morally distressing. However, in situations of scarcity, it is important to recognize that the alternative to explicit rationing is not to avoid rationing entirely, but rather to do so in an implicit or haphazard way. A system of explicit rationing offers the opportunity to intentionally support ethical principles and iteratively improve over time. While the barriers to a more explicit set of rules and processes guiding transplant candidate selection may be substantial, the current system of *de facto* rationing remains susceptible to racism, sexism, and other ethically unsupportable influences. This reality provides a powerful incentive to work toward a more explicit and equitable system of transplant evaluation and selection (Epstein et al. 2000; Patzer et al. 2015; Patzer and McPherson 2019). In order to build public trust, clinician leaders and policy makers should strive to be inclusive of different perspectives in the development and refinement of a system of selection. Efforts can include honest discussion around both the value and draw-backs or harms of a system of allocation and also emphasize a commitment to improve over time.

## CONCLUSION

The use of adherence to medical recommendations as a criterion for kidney transplant candidate selection is intended to maximize the aggregate benefit of a scarce supply of deceased donor kidney transplants. However, evidence that this criterion effectively supports this goal is equivocal and the practice may impinge on other ethical principles of selection including supporting equity and respect for patient autonomy. Heterogeneity and lack of transparency in how an adherence criterion is applied does not support principles of procedural justice.

We have suggested several strategies to strengthen the ethical foundation of an adherence assessment as a part of transplant evaluation. Entirely excluding adherence in this assessment could result in harm to patients with extreme barriers to self-care to the extent that they are unlikely to actually personally benefit from kidney transplant, but a minimalist nonadherence criterion could be defined to apply only in these rare circumstances. Alternatively, a standardized criterion for broader adherence behaviors could be implemented in a way that is agreed upon by the community of patients affected by this selection process. We believe that this normative analysis most strongly supports this final approach; however, development and implementation would take time. In the interim, a minimalist adherence selection criterion could be implemented to improve equity and respect for patient autonomy in the transplant selection process compared with continuing with the status quo. Successful adoption of any of these approaches would benefit from engagement with patients, families, and communities and endorsement by regulatory bodies, national transplant professional societies, and local transplant center clinician leaders.

Ethical conflicts in the use of adherence as a criterion for transplant candidacy echo the broader challenges around developing fair systems of scarce resource allocation. While revisions to the current system may not be easy, these efforts respect the increasing calls from patients, families, and patient and professional organizations to improve fairness and access to a valuable treatment option for all patients with kidney failure (Rosas and Reid 2025).
